# Unique presentation of a novel gain-of-function mutation in *MTOR*

**DOI:** 10.1016/j.gendis.2024.101405

**Published:** 2024-09-02

**Authors:** Samira Slimani, Alex G.I. Gagnon, Simon V. Schreiber, Nicolas A.D. Bergeron, Ludwig Haydock, Sébastien Labonté, Marc-Étienne Huot, Alexandre P. Garneau, Guillaume Canaud, Paul Isenring

**Affiliations:** Centre de recherche du CHU de Québec at the L’Hôtel-Dieu de Québec, Services de néphrologie, pathologie et cancérologie, Département de médecine, Université Laval, Québec G1R 2J6, Canada; Centre de recherche du CHU de Québec at the L’Hôtel-Dieu de Québec, Services de néphrologie, pathologie et cancérologie, Département de médecine, Université Laval, Québec G1R 2J6, Canada; Service de Néphrologie–Transplantation Rénale Adultes, Hôpital Necker-Enfants Malades, AP-HP, Inserm U1151, Université Paris Cité, rue de Sèvres, Paris 75015, France; Centre de recherche du CHU de Québec at the L’Hôtel-Dieu de Québec, Services de néphrologie, pathologie et cancérologie, Département de médecine, Université Laval, Québec G1R 2J6, Canada; Service de Néphrologie–Transplantation Rénale Adultes, Hôpital Necker-Enfants Malades, AP-HP, Inserm U1151, Université Paris Cité, rue de Sèvres, Paris 75015, France; Centre de recherche du CHU de Québec at the L’Hôtel-Dieu de Québec, Services de néphrologie, pathologie et cancérologie, Département de médecine, Université Laval, Québec G1R 2J6, Canada

Smith-Kingsmore syndrome (SKS) is a very rare dominantly inherited disorder caused by a gain-of-function mutation in *MTOR* (mechanistic target of rapamycin).^1^^,^^2^ It is typically characterized by megalencephaly, developmental and intellectual delay, and distinctive facial traits. Attenuated SKS-like phenotypes from a somatic gain-of-function mutation in *MTOR* have also been reported.^3^^,^^4^ In most of these milder cases, mosaicism translated into focal brain cortical dysplasia, and in six cases, into a somewhat wider presentation such as ventriculomegaly, patchy hypomelanosis and a few additional SKS-like features (in all six cases), unilateral megalencephaly (in three), and hemihypertrophy (in one).

More than 20 mutations have been identified in germinal and somatic SKS.^1^^,^^2^ In most instances, they consist of non-conservative missense substitutions and less commonly, short deletions. They often occur in the so-called FAT protein-interacting module but can also affect the kinase domain. While they almost all differ in nature between germinal and somatic SKS, they tend to affect the proximal portion of this domain (residues 1449–1461) in the acquired forms.^1^, ^2^, ^3^, ^4^ As it stands, the mechanisms of enzyme overactivation remain unsettled.

This letter is concerned with the in-depth molecular investigation of a clinically unique form of somatic *MTOR*-related condition that presented as an isolated 19 × 7 × 4 cm adipovascular mass along the medial aspect of the right knee (Fig. 1A, B) and was shown to arise from a previously unreported activating mutation in *MTOR*.

**Figure 1:**
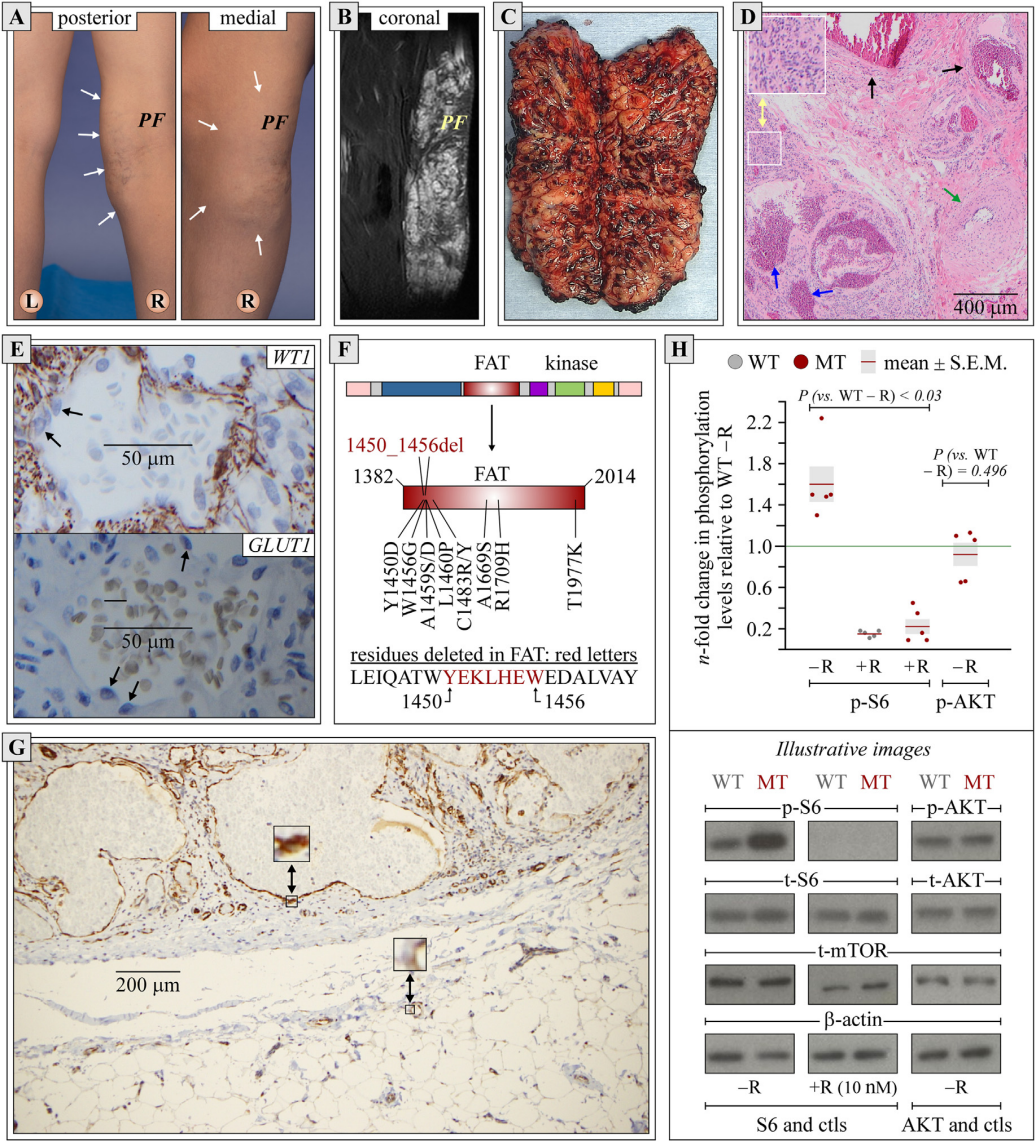
Clinical and molecular data. See text and supplementary material for methods used. Note that specimens in panels D, E, and G were analyzed by light microscopy. **(A)** External phenotype. Localization of mass is delimited by arrows. L, left; PF, popliteal fossa; R, right. **(B)** MRI (STIR modality). Large multiloculated vascular mass is revealed by whitish signals. It was embedded in a bulky lipomatous lesion by T2-weighted imaging (not shown). **(C)** Macroscopic appearance post-excision. Partially bisected mass is shown to enclose adipose tissues interspersed with serpiginous vessel-like structures. **(D)** Histopathological findings by hematoxylin and eosin staining. The mass is seen to consist of a benign hemangioma with capillary proliferation (yellow arrows) and venous, arterial, and cavernous features (black, green, and blue arrows, respectively). **(E)** Expression of WT1 and GLUT1 by immunohistochemistry. Endothelial cells were found to test negative for either protein as expected (see arrows). **(F)** Deletion identified in FAT domain. Localization of previously reported gain-of-function mutations in this domain is also shown. **(G)** Expression of phosphorylated S6RP (p-S6) by immunohistochemistry. As pointed out by arrows, the cytosol of endothelial cells exhibits intense and diffuse staining in hemangioma (upper section of the panel) but weak staining in an adjacent area of normal tissue (bottom section of the panel). **(H)** The western blots depicting the effect of MTOR^Y1450_W1456del^ (±rapamycin) on the expression of p-S6 *vs.* total S6RP (t-S6) and p-AKT *vs.* t-AKT in HEK-293 cells. Data in the upper section consist of normalized densitometry values (p-S6/t-S6/β-ACTIN and p-AKT/t-AKT/β-ACTIN). They are shown as dots (one/experiment) and expressed as mean ± standard error of the mean among five experiments. Differences between means were analyzed by paired student's *t*-tests and considered statistically different at *P* values < 0.05. Data in the bottom section correspond to illustrative autoradiograms. Ctls, controls; MT, mutant, R, rapamycin; WT, wildtype.

The tumor had been developing since age 5 in the absence of other abnormalities or features as determined through physical examination and a total body MRI. It eventually led to local discomfort and varices in the overlying skin (Fig. 1C) such that it was excised at age 13. It was shown then to consist of an admixture of adipose tissue and multiple benign hemangiomatous lesions (Fig. 1C–E) that extended across all margins but were interspersed with healthy-appearing tissues.

The hemangioma was investigated further for the presence of somatic mutations by analyzing 523 cancer genes (listed in the supplemental material, section I) through next-generation exome sequencing at a minimal coverage depth of 433 reads and minimal allelic frequency filter of 1%. The variant uncovered in *MTOR* was categorized as *Tier III* or of unknown significance but suspected on our part to be gain-of-function. It corresponded to a 21-bp deletion (4348_4368) near the 5′ end of the FAT-encoding domain (Fig. 1F) and was found to occur at an allelic frequency of 5.1%. No other somatic variants could be identified and tumor mutation burden as well as microsatellite instability were low.

The variant identified was later tested for its functional impact on MTOR based on S6RP (S6 ribosomal protein) phosphorylation levels in both the hemangioma and HEK-293 cells (see methods in supplementary material, section II to IV). Through these studies, MTOR activity was seen to be much higher in the endothelial cells of malformed vessels than in those of normal vessels (Fig. 1G) and higher in MTOR^Y1450_W1456del^-expressing HEK-293 cells than in MTOR^WT^-expressing HEK-293 cells while remaining rapamycin-sensitive (Fig. 1H). No differences could be seen in AKT (protein kinase B) phosphorylation levels between the cell lines and no differences either in total MTOR, S6RP, or AKT expression levels.

Through this letter, we have thus reported unprecedented findings in the field of *MTOR*-related disorders, *i.e.*, a clinical presentation that consisted of a confined adipohemangioma and a previously unreported MTORC1 (MTOR complex 1)-activating deletion in the enzyme (Fig. 1F). It is of note that missense mutations in the FAT-encoding domain of *MTOR* have been hypothesized to act by destabilizing an autoinhibitory domain 5. That a theoretically more disruptive 7-residue deletion would still lead to enhanced enzyme function provides much stronger evidence in support of this possibility and highlights the importance of MTORC1 in adipogenesis quite eloquently.

In conclusion, the investigation conducted has shown that the clinical presentation of a gain-of-function mutation in *MTOR* could be considerably more varied than recently reported and would then be inadequately referred to via the eponym SKS. The investigation conducted has also allowed us to identify a potentially actionable gene defect and to gain insight into the mechanisms through which mutations in the FAT domain could cause induction of the enzyme.

## Ethics declaration

The study was conducted in accordance with the Declaration of Helsinki, was authorized by the VRR de l'Université Laval under protocol SIRUL129016, and was submitted for publication after written informed consent of the patient affected by the hemangioma.

## Conflict of interests

The authors have no conflict of interests to declare.

## Funding

This work was supported by the Canadian Institute of Health and Research. The authors are grateful to Miss Marie-Jeanne Fiola and Miss Daniele Veillette for superb technical assistance.

## Data availability

The data will be made available upon request to the corresponding author.

## CRediT authorship contribution statement

**Samira Slimani:** Formal analysis, Methodology. **Alex G.I. Gagnon:** Formal analysis, Methodology. **Simon V. Schreiber:** Formal analysis, Methodology. **Nicolas A.D. Bergeron:** Methodology. **Ludwig Haydock:** Data curation, Formal analysis, Investigation, Validation. **Sébastien Labonté:** Formal analysis. **Marc-Étienne Huot:** Data curation, Formal analysis, Methodology, Validation. **Alexandre P. Garneau:** Data curation, Formal analysis, Validation. **Guillaume Canaud:** Data curation, Formal analysis, Validation. **Paul Isenring:** Conceptualization, Data curation, Formal analysis, Funding acquisition, Investigation, Methodology, Resources, Supervision, Validation, Writing – review & editing.
