# Supplementary material for: Unique presentation of a novel gain-of-function mutation in MTOR
Source: Genes Dis. 2024 Sep 2;12(2):101405. doi: 10.1016/j.gendis.2024.101405 (PMC11585748; doi:10.1016/j.gendis.2024.101405)
Supplement: Multimedia component 1 [file mmc1.docx]

**Detailed Methods – Unique presentation of a novel gain-of-function mutation in *MTOR***

Section I. Genes tested

*ABRAXAS1, AKT1, AKT2, AKT3, ALK, APC, AR, ARID1A, ATM, ATR, BAP1, BARD1, BCL2, BCL6, BRAF, BRCA1, BRCA2, BRIP1, BTK, CARD11, CCND1, CCND2, CCNE1, CD79A, CD79B, CDH1, CDK12, CDK4, CDK6, CDKN2A, CEBPA, CHEK1, CHEK2, CREBBP, CSF1R, CTNNB1, DDR2, DNMT3A, EGFR, EP300, ERBB2, ERBB3, ERBB4, ERCC1, ERCC2, ERG, ESR1, EZH2, FANCI, FANCL, FBXW7, FGF1, FGF10, FGF14, FGF2, FGF23, FGF3, FGF4, FGF5, FGF6, FGF7, FGF8, FGF9, FGFR1, FGFR2, FGFR3, FGFR4, FLT1, FLT3, FOXL2, GEN1, GNA11, GNAQ, GNAS, HNF1A, HRAS, IDH1, IDH2, INPP4B, JAK2, JAK3, KDR, KIT, KMT2A, KRAS, MAP2K1, MAP2K2, MCL1, MDM2, MDM4, MET, MLH1, MLLT3, MPL, MRE11, MSH2, MSH3, MSH6, MTOR, MUTYH, MYC, MYCL, MYCN, MYD88, NBN, NF1, NOTCH1, NOTCH2, NOTCH3, NPM1, NRAS, NRG1, PALB2, PDGFRA, PDGFRB, PIK3CA, PIK3CB, PIK3CD, PIK3CG, PIK3R1, PMS2, PPP2R2A, PTCH1, PTEN, PTPN11, RAD51, RAD51B, RAD51C, RAD51D, RAD54L, RB1, RET, RICTOR, ROS1, RPS6KB1, SLX4, SMAD4, SMARCB1, SMO, SRC, STK11, TERT, TET2, TP53, TSC1, TSC2, VHL, XRCC2.*

Section II. Strategy used to generate and characterize the cell lines

*A) Constructs.* An MTOR^WT^-enclosing plasmid was obtained from Addgene. This plasmid is composed of the following sequence from 5’ to 3’: the FLAG epitope, the coding sequence of MTOR and the vector pCDNA3 vector. It was transfected as is in cell lines (see below) or used as a template to remove 21 base pairs from the MTOR cDNA sequence (base pairs 4340 to 4370 → residues 1450 to 1456) using the Q5^®^ Site-Directed Mutagenesis Kit from New England Biolabs along with the forward oligonucleotide (written 5’ to 3’) GAGGATGCCCTTGTG and backward oligonucleotide (also written 5’ to 3’) CCAGGTAGCCTGGATC.

*B) Cell line exploited.* The HEK-293 cell line was used as the expression system for the studies was purchased from ATCC and plated on 10-cm culture dishes in DMEM+, that is, DMEM added with glucose (1 g/L), L-glutamine, sodium pyruvate, 10% FBS and 1% penicillin/streptomycin.

*C) Transfections.* Expression of FLAG-TOR^WT^ or FLAG-MTOR^Y1450_W1456del^ in HEK-293 cells was achieved by adding them (while at 60% confluence in the culture dishes) with a transfection cocktail (including 1 mL opti-MEM, 14 μg DNA constructs, 28 μL P3000 and 21.7 μL lipofectamine 3000) and by maintaining them in the same media for 48 h. In some experiments, rapamycin was added during the final 4 h of this 2-d incubation period.

*D) Western blot and statistical analyses.* Cells were collected in the RIPA lysis buffer (1 mL per tissue culture dish) and the samples so-obtained loaded on SDS-PAGE electrophoresis gels (11 μg proteins per lane based on Bradford assays). Blotting was carried with PVFD membranes and protein detection with a combination of primary and secondary antibodies (see *Section IV* below with the concentrations used). Additional details appear in the figure legend of the research letter.

Section III. Histological analyses of thin paraffin-embedded tissue sections obtained from the hemangioma and transferred onto glass slides

*A) Routine staining.* Slides were allowed to dry, deparaffinized, rehydrated and H&E-stained.

*B) Immunohistochemistry.* Slides were processed through the automated FLEX-based detection system (Agilent Technologies). In this system, epitopes exposed to primary and secondary antibodies (see *Section IV*) are revealed through peroxidase staining and slides are counterstained with hematoxylin.

Section IV. Antibodies used

*A)* *Primary antibodies used*

Targets of origin concentration dilution final company from

antibodies of of stock used in concentration which antibody was

used antibody (μg/mL) study (ng/mL) purchased

S6RP mouse 4 1:1000 4 Cell signaling technology

phospho-S6RP (WB)^1^ rabbit 24 1:1000 24 Cell signaling technology

phospho-S6RP (IHC)^1^ rabbit 24 1:100 240 Cell signaling technology

pan-AKT rabbit 35 1:1000 35 Cell signaling technology

pan-phospho-AKT^2^ rabbit 91 1:1000 91 Cell signaling technology

FLAG epitope mouse 4600 1:4000 1150 Sigma

β-ACTIN mouse 1000 1:1000 1000 Sigma

WT1 (IHC) mouse 1000 1:400 2500 Thermo Fisher Scientific

GLUT1 (IHC) rabbit 240 1:1000 240 Abcam

^1^ S6RP^S240/S244^; ^2^ AKT^S473^; IHC, immunohistochemistry; WB, Western blot analysis

*B)* *Secondary antibodies used*

Name of origin target dilution company from

antibodies of antibodies used in which antibody was

used antibody probed study purchased

HRP-linked sheep anti-S6RP 1:2000 Fisher scientific

anti-IGG sheep anti-FLAG 1:5000 Fisher scientific

sheep anti-β-ACTIN 1:5000 Fisher scientific

HRP-linked donkey anti-phospho-S6RP 1:5000 Fisher scientific

anti-IGG donkey anti-AKT 1:5000 Fisher scientific

donkey anti-phospho-AKT 1:5000 Fisher scientific

FLEX rabbit anti-phospho-S6RP – Agilent technologies

system mouse anti-WT1 – Agilent technologies

mouse anti-GLUT1 – Agilent technologies

HRP, horseradish peroxidase
